# Control of Trachoma from Achham District, Nepal: A Cross-Sectional Study from the Nepal National Trachoma Program

**DOI:** 10.1371/journal.pntd.0004462

**Published:** 2016-02-12

**Authors:** Bidya Prasad Pant, Ramesh C. Bhatta, J. S. P. Chaudhary, Suresh Awasthi, Sailesh Mishra, Shekhar Sharma, Puja A. Cuddapah, Sarah E. Gwyn, Nicole E. Stoller, Diana L. Martin, Jeremy D. Keenan, Thomas M. Lietman, Bruce D. Gaynor

**Affiliations:** 1 Geta Eye Hospital, Dhangadhi, Nepal; 2 Nepal Netra Jyoti Sangh, Tripureswor, Kathmandu, Nepal; 3 F.I. Proctor Foundation, University of California, San Francisco, San Francisco, California, United States of America; 4 Division of Parasitic Diseases and Malaria, Centers for Disease Control and Prevention, Atlanta, Georgia, United States of America; 5 Department of Ophthalmology, University of California, San Francisco, San Francisco, California, United States of America; 6 Department of Epidemiology & Biostatistics, University of California, San Francisco, San Francisco, California, United States of America; University of Cambridge, UNITED KINGDOM

## Abstract

**Background:**

The WHO seeks to control trachoma as a public health problem in endemic areas. Achham District in western Nepal was found to have TF (trachoma follicular) above 20% in a 2006 government survey, triggering 3 annual mass drug administrations finishing in 2010. Here we assess the level of control that has been achieved using surveillance for clinical disease, ocular chlamydia trachomatis infection, and serology for antibodies against chlamydia trachomatis protein antigens.

**Methods:**

We conducted a cross-sectional survey of children aged 1–9 years in communities in Achham District in early 2014 including clinical examination validated with photographs, conjunctival samples for *Chlamydia trachomatis* (Amplicor PCR), and serological testing for antibodies against chlamydia trachomatis protein antigens pgp3 and CT694 using the Luminex platform.

**Findings:**

In 24 randomly selected communities, the prevalence of trachoma (TF and/or TI) in 1–9 year olds was 3/1124 (0.3%, 95% CI 0.1 to 0.8%), and the prevalence of ocular chlamydia trachomatis infection was 0/1124 (0%, 95% CI 0 to 0.3%). In 18 communities selected because they had the highest prevalence of trachoma in a previous survey, the prevalence of TF and/or TI was 7/716 (1.0%, 95% CI 0.4 to 2.0%) and the prevalence of ocular chlamydia trachomatis infection was 0/716 (0%, 95% CI 0 to 0.5%). In 3 communities selected for serological testing, the prevalence of trachoma was 0/68 (0%, 95% CI 0 to 5.3%), the prevalence of ocular chlamydia trachomatis infection was 0/68 (0%, 95% CI 0 to 0.5%), the prevalence of antibodies against chlamydia trachomatis protein antigen pgp3 was 1/68 (1.5%, 95% CI 0.04% to 7.9%), and the prevalence of antibodies against chlamydia trachomatis protein antigen CT694 was 0/68 (0%, 95% CI 0 to 5.3%).

**Conclusion/Significance:**

This previously highly endemic district in Nepal has little evidence of recent clinical disease, chlamydia trachomatis infection, or serological evidence of trachoma, suggesting that epidemiological control has been achieved.

## Author Summary

Trachoma is the most common cause of blindness from a bacterial infection in the world. It is caused by the bacterium *Chlamydia trachomatis*. The WHO (World Health Organization) seeks to control this disease worldwide using SAFE which includes mass antibiotic administrations (MDA) with azithromycin. The Nepal National Trachoma Program was started in 2002 with the goal of controlling trachoma as a public health problem. We did a survey in Achham District in early 2014 and found the prevalence of clinical signs of trachoma, chlamydia trachomatis infection and antibodies against chlamydia trachomatis were all below 1% in 1–9 year olds. Trachoma has been controlled and is no longer a public health problem in Achham district Nepal.

## Introduction

The Nepal National Trachoma Program (NTP) was initiated in 2002 with the goal of controlling trachoma in Nepal by 2014.[1] The WHO SAFE (Surgery, Antibiotics, Facial Cleanliness, Environmental Improvements) strategy was employed including mass drug administration (MDA) with azithromycin in 17 districts between 2002 and 2012. Population-based surveys of trachoma were performed by Nepal Netra Joyti Sangh (NNJS) workers to evaluate the Nepal program and to identify Districts where further treatment was recommended. Achham District, which was surveyed in 2006, was recognized as an area with the highest prevalence of TF in Nepal (> 20%) among children 1–9 years, triggering 3 MDAs distributed in 2008, 2009 and 2010 with over 500,000 treatments.[1] A follow-up survey in September 2012 determined TF prevalence in Achham was 5.9%, still the highest prevalence among Nepali Districts[1] triggering an additional round of treatment offered in 2014 in accordance with the WHO recommendations prior to our measurements.

We aimed to determine if control had been achieved in the clinical signs of trachoma by examination with certified graders (validated with photographs), in infection with *Chlamydia trachomatis* by PCR, and in antibody responses to *C*. *trachomatis* protein antigens pgp3 and CT694 using serological testing, through population-based cross-sectional measurements in Achham District in early 2014 after repeated rounds of drug administrations had been offered.

## Methods

### Study population

Nepal is divided into 75 Districts and this study took place in Achham District, Seti Zone. Districts are divided into Village Development Committees (VDCs) based on population size for the purpose of delivering health care and other services. Achham District is divided into 75 VDCs and inclusion criteria for this study was accessibility less than a 2-hour walk from a vehicle-accessible road. Each VDC is further divided into 9 wards referred to as communities in this manuscript.

### Community randomization

**Fig 1:**
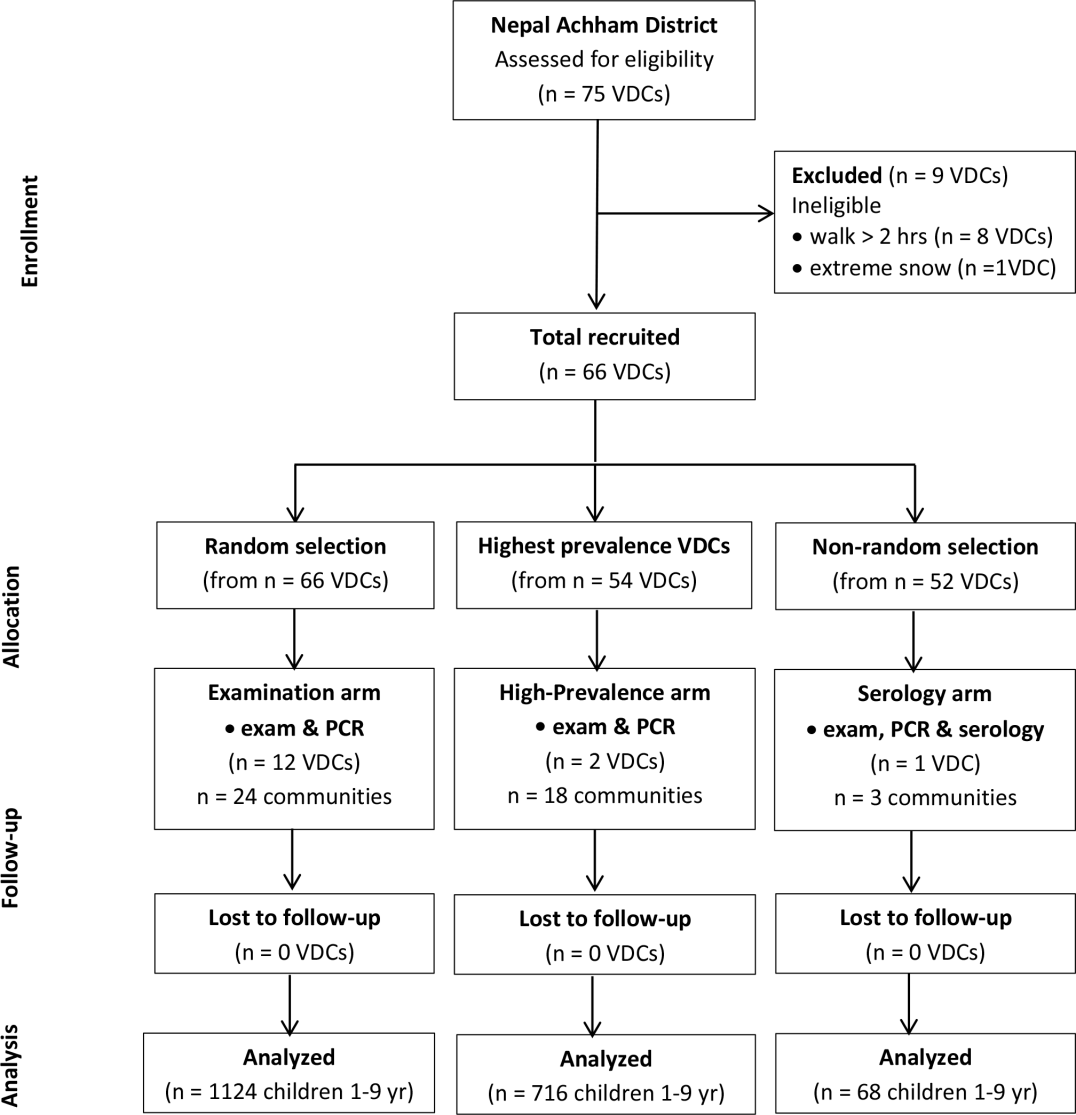
Participant flow.

In Achham District, 12 VDCs and 2 communities per VDC were randomly selected (n = 24 communities) for the Examination arm where examination and PCR were performed in January 2014 (Fig 1). For more detailed assessment of disease prevalence, 2 VDCs (18 communities) which were identified as high trachoma-prevalence areas (TF > 20%) by NNJS in the September 2012 survey, were included in the High Prevalence arm where examination and PCR were performed in January 2014. Finally, when serological testing for *C*. *trachomatis* became available to us in May 2014, another VDC (with 3 communities) conveniently located was chosen for serology testing in addition to examination and PCR in the Serology arm. Randomization of the 12 VDCs in the Examination arm was performed using Microsoft Excel (Version 2010, RAND and Sort functions).

A current population census was unavailable so household heads and guardians were asked to bring children to a communal space such as a neighborhood school, community center, or other area well known to villagers with open land where examination and sample collection could be performed. For each household included, the name of the household head was recorded along with the age and gender of each child aged 1–9 years. Written informed consent was obtained in the local language by a parent or guardian prior to examination and sample collection.

### Grading of clinical trachoma

Clinical grading of trachoma (TF and/or TI) of the right everted superior tarsal conjunctiva was done using the WHO simplified grading system.[2] Clinical graders were allowed to grade for the study if they attained a chance corrected agreement *k* ≥ 0.6 with an experienced grader (BDG, JDK or TML) over the scoring signs of clinically active trachoma (TF and TI) during validation exercises with 200 previously graded photographic examples from children 1–9 years. Following grading and prior to any sample collection, at least 2 photographs of the upper tarsal conjunctiva were taken with a Nikon D-series camera and a Micro Nikkor 105 mm f/2.8 lens (Nikon, Tokyo, Japan) for subsequent auditing.

### *Chlamydia trachomatis* detection with PCR

After clinical grading and photography, examiners wearing latex gloves passed a Dacron swab firmly three times over the right upper tarsal conjunctiva, rotating 120 degrees between each pass as previously described.[3,4] Field workers changed gloves between all participants or if there was suspicion of contamination at any time. Swabs were placed in a sterile micro tube with the help of another gloved examiner to minimize contamination and placed at 4°C within 10 hours prior to shipping to University of California San Francisco on icepacks where they were stored at -80°C until processing.[5] Samples were pooled for processing to save time and cost as previously described,[6] and the COBAS Amplicor PCR assay (Roche Diagnostics, Branchburg, NJ, USA) for nucleic acid amplification testing (NAAT) was used to detect *C*. *trachomatis* DNA. Any equivocal pools were separated and tested as individual samples.

To test for contamination, 5 participants were randomly selected during each community visit to receive an “air swab” where the swab was used in the usual fashion but passed within 5 cm without contacting the participant and processed with other samples by masked lab personal as previously described.[7] Positive and negative controls for *C*. *trachomatis* were included in laboratory analyses.

### Serological testing for *C*. *trachomatis* specific antibodies

In the 3 communities in the Serology arm, 10μL of blood was collected from enrolled study participants after a finger prick onto extensions of filter paper (TropBio Pty Ltd, Townsville, Queensland, Australia) calibrated to hold 10 μL of blood. The blood was, air dried and filter papers placed in individual zip-lock bags before freezing at -20°C. Dried blood spots were shipped to the Centers for Disease Control and Prevention in Atlanta GA, USA and were analyzed individually for detection of IgG antibodies against *C*. *trachomatis* derived protein antigens pgp3 and CT694, on the Luminex platform (Luminex Corporation, Austin, TX) as previously described.[8,9] Cutoffs for positivity were based off receiver operating characteristics from a set of sera from 122 children < 6 years old from the United States as a negative population and a set of 10 Tanzanian children with ocular swabs positive for *C*. *trachomatis* nucleic acid. The cutoff for pgp3 was 882 and for CT694 was 137 (arbitrary units detected median fluorescent intensity [MFI]).

### Ethics statement

Ethical approval for this study was obtained from the Ethical Review Board of the Nepal Health Research Council (Ref No. 63/2014) and the Committee for Human Research of the University of California, San Francisco (IRB # 13–10961, Ref No. 067007). Oral consent was obtained from the community leaders, and written (thumbprint) informed consent was obtained from the child’s parent or guardian in the local language prior to examination and sample collection. The use of thumbprint consent instead of a written signature was approved by the Ethical Review Board of the Nepal Health Research Council and the Committee for Human Research of the University of California, San Francisco. The study was carried out in accordance with the Declaration of Helsinki.

## Results

The participant flow for the study in Achham District is shown in Fig 1. Twelve VDCs that met inclusion criteria were randomly selected and included in the Examination arm, two VDCs were included in the High Prevalence arm, and one VDC was included in the Serology arm. Baseline characteristics were similar in Examination, High-Prevalence and Serology arms (Table 1). The results for clinical trachoma examination, chlamydia trachomatis infection and antibodies against chlamydia trachomatis protein antigens in the Examination, High-Prevalence and Serology arms are shown in Table 2.

**Table 1:** Baseline characteristics of communities in examination, high-prevalence and serology arms.

	Examination	High-Prevalence	Serology
**Communities**	24	18	3
**Participants, 1–9 years**	1124	716	68
**Age, years**^a^	5.1 (4.9 to 5.2)	5.1 (4.9 to 5.3)	5.2 (4.6 to 5.8)
**Gender, female**^a^	50.1% (47.7 to 53.6%)	49.9% (41.1 to 53.6%)	44.1% (32.0 to 56.7%)

^a^Community means with 95% CIs.

**Table 2:** Prevalence of trachoma, infection and antibodies against *C*. *trachomatis* protein antigens in examination, high-prevalence and serology arms. Community means with 95% CIs.

	Examination	High-Prevalence	Serology
**Trachoma**^a^	0.3% (0.1 to 0.8%)	1.0% (0.4 to 2.0%)	0% (0 to 5.3%)
***C*. *trachomatis***^b^	0% (0 to 0.3%)	0% (0 to 0.5%)	0% (0 to 0.5%)
**pgp3**^c^			1.5% (0.04 to 7.9%)
**CT694**^c^			0% (0 to 5.3%)

^a^TF and/or TI using WHO system.

^b^Amplicor PCR

^c^*C*. *trachomatis* protein antigens.

### Clinical signs of trachoma, *C*. *trachomatis* infection and serology

In the Examination arm, community prevalence of clinical trachoma in 1–9 year olds was 3/1124 (0.3%, 95% CI 0.1 to 0.8%), and prevalence of ocular *C*. *trachomatis* infection by PCR was 0/1124 (0%, 95% CI 0 to 0.3%). In the High-Prevalence arm, community prevalence of clinical trachoma in 1–9 year olds was 7/716 (1.0%, 95% CI 0.4 to 2.0%), prevalence of ocular *C*. *trachomatis* infection by PCR was 0/716 (0%, 95% CI 0 to 0.5%). In the Serology arm, community prevalence of clinical trachoma was 0/68 (0%, 95% CI 0 to 5.3%), prevalence of ocular *C*. *trachomatis* infection by PCR was 0/68 (0%, 95% CI 0 to 0.5%), prevalence of antibodies against chlamydia trachomatis protein antigen pgp3 was 1/68 (1.5%, 95% CI 0.04 to 7.9%), and prevalence of antibodies against chlamydia trachomatis protein antigen CT694 was 0/68 (0%, 95% CI 0 to 5.3%). The antibody-positive sample had a signal of 4782 MFI (from a scale of 35,000). The 143 field control swabs were negative for chlamydia trachomatis by PCR.

## Discussion

We found clinical trachoma, ocular chlamydia trachomatis infection by PCR and anti-chlamydia trachomatis antibody responses in 1–9 year olds have been controlled in Achham District as measured in this cross-sectional assessment in January and May 2014.[10,11] Serological testing for chlamydia trachomatis is a new alternative for trachoma surveillance and may be useful tool particularly in areas where chlamydia trachomatis and clinical trachoma are controlled.[8,9]

The First and Second meetings in 1997 and 1998 of the WHO Global Alliance for the Elimination of Trachoma suggested that national programs may be necessary to control trachoma as a public health problem.[12,13] The government of Nepal established the National Trachoma Program in 2002 and implemented MDAs with azithromycin in 17 districts thought to be at high risk for trachoma to achieve this goal. The trachoma prevalence estimate for Achham District was 5.9% in 2012 triggering an additional round of mass antibiotic treatment offered in 2014, prior to our measurements. The WHO recommendation recently updated should trigger reassessment of Achham District following treatment before additional treatments are offered.[14] This will maximize the effect of antibiotics in short supply and minimize adverse events.

The WHO advises hygiene and environmental improvements to prevent trachoma from returning after antibiotics are discontinued, but there is no evidence any particular intervention is beneficial. In this cross-sectional assessment of Achham District in 2014, we found clinical trachoma, chlamydia trachomatis infection, and antibodies against chlamydia trachomatis protein antigens were undetectable or present at only low levels. Trachoma is controlled in this district and even the loftier goal of local elimination of *C*. *trachomatis* infection has been achieved.

This study has limitations which may affect generalizability. The 66 VDCs were randomly selected in the Examination arm, but 2 VDCs in the High-Prevalence arm were selected based on a government survey from September 2012 introducing potential selection bias overestimating prevalence. Inaccurate grading is always a concern with clinical trachoma work. The graders in Nepal were probably different in the 2006 and 2012 assessments introducing variation. To maximize consistency, all graders who took part were certified with *k* ≥ 0.6 agreement with experienced graders prior to participating and all individuals examined in 2014 had a photograph taken that was subsequently audited by experienced graders. The study only included 1–9 year olds and adults may harbor disease that was missed. However, this is unlikely because the reservoir of trachoma is almost exclusively in preschool children shown in numerous studies.[9,15,16] The negative serology for chlamydia trachomatis was surprising in this previously endemic region, but chlamydia trachomatis antibodies might be detected in adults previously infected who were not sampled here.[9] Note the small serological sample size of 68 may limit generalizability.

Conjunctival samples were pooled into pools of five for PCR to save time and cost as previously described.[6,17] Serological samples were not pooled and were tested as individual samples. The pooling process may have interfered with detection of positive chlamydia trachomatis samples but any equivocal pools were separated and tested as individual samples making this less likely Pooling could in theory reduce the concentration below a detectability threshold, although we have unpooled 100s of pools from previous studies and found that a negative pool with a positive individual sample is no more common than an individual test being positive and then negative when retested—that is, the discrepancy is within the repeatability limit of the test, rather than a dilutional effect.[3,4,18,19,20,21] Also, we’ve been just as likely to find a positive pool and a negative individual test.

Some researchers and program managers believe that children in disadvantaged families are more likely to harbor infection but less likely to be brought for examination and treatment, resulting in an underestimation of trachoma prevalence. However, in a study in Niger where trachoma is highly endemic, we found preschool children who have ocular *C*. *trachomatis* have higher odds of presenting early for examination than those who are not infected.[7] Inclusion criteria for VDCs was accessibility less than 2 hours walk from a vehicle-accessible road. This may have introduced bias if less accessible areas are more or less likely to harbor trachoma.

There are various reasons which explain the marked drop in trachoma in Achham District from 2006 to 2014. The Nepal Trachoma Program distributed mass antibiotics in 2008, 2009, and 2010 and implemented face washing and environmental improvements during that time as well. We are cautious in attributing the reduction in trachoma in western Nepal solely to the National Program and must consider other possibilities. In a previous study in 52 villages in Kailali and Konchapur Districts adjacent to Achham District in Western Nepal from 1998 to 2001, we observed that 21% of the reduction in trachoma could be attributed to secular trends rather than programmatic antibiotic distributions.[22] Secular trends in trachoma have also been seen in the Gambia, Malawi, and European countries even without antibiotic distributions.[23,24,25] Much of the reduction of trachoma in Nepal may be due to a secular trend in the area rather than a specific effect of the trachoma control program.

Control of trachoma has been the WHO goal, as the prevalence of blindness is probably negligible when the clinical signs of infection are brought to a low level. Currently, the WHO aim for control is less than 5% district prevalence of TF in children.[26] Achham Nepal has achieved this target. In fact, we found no evidence of current infection and minimal evidence of recent infection by serology. Thus, true elimination of infection may have been achieved here and might be a reasonable goal in similar areas. Other studies have shown significant reduction and elimination of clinical trachoma and chlamydia trachomatis infection in one or even in multiple communities.[27,28,29,30] However, trachoma may return to previously affected communities by contamination from adjacent areas or from migration when antibiotics are discontinued.[3,31]. Here we demonstrate results consistent with absence of signs of clinical trachoma (verified with photographs), chlamydia trachomatis infection (Amplicor PCR), and antibodies against chlamydia trachomatis protein antigens (Luminex) in an entire district. In once severely affected districts like this, elimination of infection may be a realistic goal—not merely control of the clinical signs of trachoma to a low level.

## Supporting Information

S1 Checklist Strobe Checklist for observational studies. (DOCX)
